# Recent Studies on Hydrogels Based on H_2_O_2_-Responsive Moieties: Mechanism, Preparation and Application

**DOI:** 10.3390/gels8060361

**Published:** 2022-06-08

**Authors:** Weihua Song, Jipeng You, Yuangong Zhang, Qi Yang, Jin Jiao, Hailei Zhang

**Affiliations:** 1 Affiliated Hospital of Hebei University, Baoding 071000, China; hdfysongweihua@163.com (W.S.); doctoryoujipeng@126.com (J.Y.); hdfsyangqi@163.com (Q.Y.); jiaozijin0011@163.com (J.J.); 2 College of Chemistry and Environmental Science, Hebei University, Baoding 071002, China; zhangyuangong@hbu.edu.cn

**Keywords:** stimuli-responsive, drug delivery, halloysite nanotube, biomedical, self-crosslinking, difunctional crosslinkers

## Abstract

H_2_O_2_ is essential for cellular processes and plays a vital role in the regulation of cell signaling pathways, which can be viewed as a warning signal for many kinds of disease including cancer, cardiovascular disease, reproductive abnormalities, diabetes, and renal failure. A H_2_O_2_-responsive hydrogel (H_2_O_2_-Gel) is a promising candidate for biomedical applications because of its good biocompatibility, similarity to soft biological tissues, ease of preparation, and its ability to respond to H_2_O_2_. In this study, the H_2_O_2_-responsive moieties used to fabricate H_2_O_2_-Gels were reviewed, including thioethers, disulfide bonds, selenides, diselenium bonds, diketones, boronic, and others. Next, the preparation method of H_2_O_2_-Gel was divided into two major categories according to their reaction mechanisms: either self-crosslinking or mechanisms entailing the addition of difunctional crosslinkers. Last, the applications of H_2_O_2_-Gels were emphasized, which have been viewed as desirable candidates in the fields of drug delivery, the detection of H_2_O_2_, glucose-responsive systems, ROS scavengers, tissue engineering, and cell-encapsulation.

## 1. Introduction

In recent years, relevant researchers have developed a variety of stimulation mechanisms by investigating the changes in the microenvironment of the human body [1,2,3]. Following this research, quite a number of stimuli-responsive materials have been developed with sensibility to acidity [4], alkali [5], temperature [6], mechanical force [7], magnetic fields [8], ultrasound [9], reactive oxygen species (ROS) [10], etc. ROS are emerging as critical signaling molecules which are abundant in the human microenvironment [11]. The term ROS encompasses a wide range of molecules, such as hydrogen peroxide (H_2_O_2_), singlet oxygen (^1^O_2_), hydroxyl radical, superoxide, etc. [12]. The stable control of reactive oxygen species level is of great significance to maintain the normal physiological activities of human bodies [13]. The overexpression of reactive oxygen species can easily destroy the structure of many biological macromolecules, such as proteins and nucleic acids, and thus result in a variety of diseases [14]. Therefore, it is of great theoretical and practical significance to design and develop smart materials with ROS responsive behaviors.

**Scheme 1:**
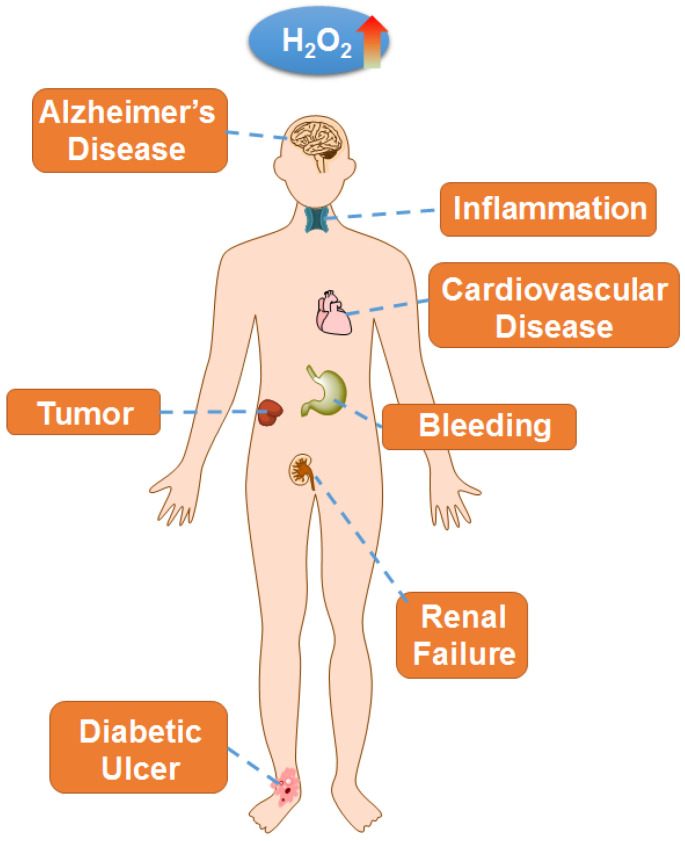
Relation between the over expression of H_2_O_2_ and related diseases.

H_2_O_2_ is a representative case of ROS that is particularly important in the physiological regulation in organisms [15]. It plays an important role in cell signal transduction, differentiation, and proliferation, and also plays an irreplaceable role in the diagnosis and treatment of many diseases [16]. Especially in diseases related to oxidative stress, a change in the H_2_O_2_ level is a good indication of the location of the lesion site [17]. When oxidative stress and injury occur in the body, the concentration of H_2_O_2_ is higher than the normal level [18]. Therefore, the over expression of H_2_O_2_ in vivo is directly related to inflammation, bleeding, Alzheimer’s disease, diabetes, renal function decline or tumors, and other diseases (Scheme 1) [19]. For example, the H_2_O_2_ level of tumor tissue is significantly higher than that of normal tissue. The H_2_O_2_ concentration in normal tissue usually maintains about 10^−8^ M, while the H_2_O_2_ concentration in inflammatory tissue or tumors is about dozens or even thousands of times higher than that in normal tissue [16]. Therefore, H_2_O_2_ is more suitable to serve as a pathological marker than pH, temperature, ionic charge, etc. The concentration of H_2_O_2_ is also overexpressed during the proliferation and metastasis of tumor cells. Therefore, the overexpression of H_2_O_2_ can be used to monitor changes in the microenvironment. The development of H_2_O_2_-responsive materials with high specificity, sensitivity, and accuracy is a meaningful and interesting topic, which may exhibit much more promising applications for early diagnosis than other stimuli responsive materials such as those that respond to pH, temperature, ionic charge, etc.

Until now, different types of H_2_O_2_-responsive materials have been explored, including MRI contrast agents, fluorescent sensors, transistors, drug delivery systems, mitochondria-directed tools, etc. [20,21]. Gu et al. published a review paper on the topic of H_2_O_2_-responsive materials, in which the biomedical applications were emphasized [17]. It is worth noting that hydrogels are widely used in the fields of drug delivery, tissue engineering, surgical dressing, etc. [22,23,24,25]. As a result, hydrogel based on H_2_O_2_-responsive moieties is a major kind of H_2_O_2_-responsive material [26,27] which can be defined as H_2_O_2_-responsive hydrogel (H_2_O_2_-Gel). There is a great deal of significance in performing a review of the development of H_2_O_2_-Gel and in the explication of the guidelines for designing suitable formations, which is also our interest in this study. Hydrogels are three-dimensional (3D) cross-linked polymer networks, which can absorb and retain large amounts of water [28]. Due to their flexible structure, stimuli-responsive hydrogels can perform work by converting an external stimulation into mechanical motions or chemical conversions, which can be an effective framework for soft actuators. In this review, we focus on overviewing the responsive mechanism of H_2_O_2_-responsive moieties and the applications of H_2_O_2_-Gel, especially in biomedicine. The preparation methods are also summarized. We expect to provide guidance and designate the direction of further studies on H_2_O_2_-responsive materials.

## 2. H_2_O_2_-Responsive Moieties

### 2.1. Thioether (-S-)

Thioether can be regarded as a compound analogous to ether in which the oxygen is replaced by sulfur. Thioether is commonly synthesized from potassium or sodium sulfide and halohydrocarbon and is widely distributed in proteins [29]. Benefiting from the higher atomic number of sulfur compared to oxygen, thioether usually shows higher chemical activity than ether and can be transferred into sulfoxide and sulfone with the treatment of H_2_O_2_ (Table 1) [30].

Napoli et al. investigated the mechanism for the oxidative destabilization of poly(propylene sulfide) [31]. Even at concentrations as low as 0.03-vol.% H_2_O_2_, the transformation from thioether to sulfoxide or sulfone can be achieved. The NMR shifts of the methyl group in the α and β position to sulfur were examined in detail to reveal the changes. After treating the material with H_2_O_2_, a doublet at ~1.53 ppm apparated corresponding to the methyl in β position to –SO_x_– moieties. Moreover, a high-frequency shift of the peaks corresponding to the protons in the α position to the –S– (2.65 and 2.93 ppm) was also observed. These phenomena indicate that both the sulfoxide and sulphone moieties are included in the backbone of H_2_O_2_-treated poly(propylene sulfide). Kramer and Deming further demonstrated the H_2_O_2-_sensitive mechanism of thioether, in which the thioether moiety in a pyranose derivative can be oxidized into sulphone-containing product with a high yield [32].

### 2.2. Disulfide Bond (-S-S-)

Disulfide bonds are commonly included in a variety of proteins, which can be generated from the oxidation of thiol groups. An S anion from one sulfhydryl group can act as a nucleophile and attack another sulfhydryl moiety to create a disulfide bond. Particularly, H_2_O_2_ can be used to break the S-S bond and yield corresponding thiolated compounds (Table 1). Due to the high activity of sulfhydryl groups, the degraded thiolated compounds have the capacity of forming the original disulfide-bearing substance. Therefore, the disulfide-based H_2_O_2_-responsive behavior can be regarded as a reversible process [33].

### 2.3. Selenide (-Se-)

Selenium (Se) is a nonmetallic element in the oxygen family that mainly exists in several allotropic forms. It is widely spread in volcanic areas and sulfide ores, which can be used in photocells, solar cells, and in xerography. Selenium can be bound into proteins and serves as an indispensable microelement in humans. Its chemical properties are similar to sulfur, but with a higher reactivity. As a result, the H_2_O_2_-treated selenide derivatives are usually transferred into the selenone form (Table 1).

Zhang’s group illustrated the structural change with the oxidation of a selenide block copolymer with H_2_O_2_ at 0.1%, and the oxidized residue was analyzed by XPS. The binding energy of Se 3d5’s shift from 55.9 eV to 59.5 eV was clearly observed, suggesting the higher valency of selenium which greatly matches the formation of selenone groups. The presence of O=Se=O groups can also be demonstrated based on the characteristic absorption bands at 904 and 880 cm^−1^. ^1^H-NMR and ^77^Se-NMR further confirmed the transformation from -Se- to O=Se=O groups, which shows a H_2_O_2_-responsive behavior [35].

### 2.4. Diselenium Bond (-Se-Se-)

A diselenium bond can be regarded as a linkage containing two atoms of selenium combined with an element or radical. Due to the similar chemical properties of selenium and sulfur, a diselenium bond can also be broken with treatment from H_2_O_2_. The diselenide bond has an energy of around 176 kJ/mol, indicating its easy oxidization in the presence of H_2_O_2_ while undergoing a phase transition from hydrophobic diselenide to hydrophilic selenic acid (Table 1) [41]. An attractive advantage of diselenide and disulfide bonds is their hydrolytic stability in physiological conditions [36]. Moreover, the diselenid-based H_2_O_2_-responsive behavior can also be regarded as a reversible process. The degraded hydrophilic selenic acid can rebuild the diselenium bond under ambient atmosphere.

Diselenium derivatives can be prepared by the oxidation of aryl selenols [42]. However, as aryl selenols are most conveniently prepared from the diselenides themselves, this method is not likely to have preparative value. Thus, diselenium derivatives are usually synthesized via other routes. For example, electron-deficient aryl halides can be used as substrates [43], and their reaction with the diselenide dianion gives rise to the desired molecules. Arylamines can also serve as potential precursors via the reaction of diazonium salts with sodium hydrogenselenide. Another typical route to diaryl diselenides is based on the reaction of arylaldehydes with sodium hydrogenselenide, which, after treatment with sodium borohydride and piperidine, leads to the target compounds.

### 2.5. Diketone

Benzil is a yellow crystalline containing a diketone moiety made by oxidizing benzoin [44]. Sawaki et al. have reported that benzil can transform into benzoic anhydride via a Baeyer-Villiger type reaction with H_2_O_2_ in alkaline organic solvents [39]. Then the products are usually presented in benzoic form due to hydrolysis (Table 1). However, the reported conditions were far from biological. Nagano’s group developed a simple strategy to adjust the reactivity between benzil and H_2_O_2_ based on the modification of the benzene ring [40]. Following this method, 5-benzoylcarbonylfluorescein derivatives were synthesized for the detection of hydrogen peroxide at the cellular level together with the d-PeT mechanism to control fluorescence. Moreover, other peroxalate ester-containing derivatives have been reported to show similar H_2_O_2_-responsive properties [45,46,47].

### 2.6. Boronic

The reaction between H_2_O_2_ and benzeneboronic acid in aqueous solutions can generate phenol and boric acid in high yields, which can be viewed as a homolytic orelectrophilic substitution in the benzene ring (Table 1) [48]. Generally, the rate of the reaction follows a dose-dependent manner. Moreover, in addition to benzeneboronic acid, quite a number of aryl-substituted boronic acids were developed, which can be exploited as H_2_O_2_-responsive tools with high accuracy, specificity, and sensitivity. Chang’s group proposed a strategy for the optical detection of H_2_O_2_ relying on the selective H_2_O_2_-triggered transformation of arylboronates to phenols [37]. With the H_2_O_2_-triggered hydrolytic deprotection of the boronates, our groups have developed a series of open, colored, and fluorescent products [49,50,51,52].

Interestingly, when the arylboronic acid ester is connected to a suitable linker, e.g., a typical –Ar–CH_2_–O– linkage, after the formation of a phenol derivative triggered by H_2_O_2_, a sequential self-immolative process can take place via a 1,4-/1,6 elimination reaction. This character of arylborates has enabled the development of H_2_O_2_-triggered degradable materials [38].

### 2.7. Others

To meet the increasing demand for H_2_O_2_-responsive materials and ROS scavengers, some other types of H_2_O_2_-sensitive linkages have been employed. Tellurium (Te)-containing organic compounds have gained attention because of their lower electronegativity, which is thought to be more easily oxidized by H_2_O_2_ than selenium/sulfur–containing ones [53]. Moreover, tellurium-containing compounds were evidenced to exhibit less toxicity than selenium analogues. It has been reported that the tellurium atoms in Te-containing organic compounds can be completely oxidized with 100 μM H_2_O_2_.

Peptides and enzymes have also been used to fabricate H_2_O_2_-responsive materials. Sung et al. reported a peptide oligomer as a H_2_O_2_-responsive crosslinker for long-term tissue engineering applications. The degradation can take place by reacting with 5 mM H_2_O_2_ [54,55].

Otherwise, some metallic oxides and metal salts can be used as H_2_O_2_-responsive moieties owing to their catalytic action or redox activity. For example, the catalytic reaction between the H_2_O_2_ and MnO_2_ contributes to the formation of H_2_O and O_2_ [56]. Fe(II) can react with H_2_O_2_ to generate hydroxyl radicals [57].

## 3. Preparation

Generally, hydrogels fall into two categories [58,59,60]:

(i) Chemical gel: Hydrogels that are covalently cross-linked networks in which the equilibrium swelling state depends on the crosslink density and interaction parameters [61,62];
(ii) Physical gel: The networks are constructed by noncovalent linkages, e.g., hydrogen bonding, ionic bonding, hydrophobic interactions, molecular entanglements, etc. These interactions are reversible.

As for H_2_O_2_-responsisve hydrogels, the responsiveness is mainly based on the linkages summarized in the abovementioned section. Such responsiveness is quite different from a response to temperature, pH, or electric fields, and the latter of these is mainly presented in physical gels. As a result, H_2_O_2_-responsisve hydrogels are mainly presented as chemical gels in the literature, in which a chemical cross-linking approach is usually involved. The cross-linking approach can be classified into two generic groups.

### 3.1. Self-Crosslinking

For this case, the H_2_O_2_-Gels were usually prepared based on the self-crosslinking behavior of the polymeric chains without the addition of any other crosslinkers. The H_2_O_2_-sensitive moieties, e.g., sulfydryl, selenol, phenol, etc., are mainly incorporated in the polymeric chains as pendent or terminal groups which can be further involved in the condensation reactions to achieve the three-dimensional structures.

Wan’s group synthesized a sulfydryl-bearing oligomer (*o*-DHLA) by treating dihydrolipoic acid with equimolar 2,2-dimethoxypropane under the catalysis of *p*-toluenesulfonic acid [63]. The obtained *o*-DHLA was achieved with sulfydryl units as terminal groups, the latter of which can react with each other to yield –S–S– containing microgels with the addition of polyvinyl alcohol. The formation of an –S–S– linkage makes the obtained microgels desirable H_2_O_2_–responsive drug delivery systems. Yang’s group employed cysteine-containing keratin as the base material, in which the sulfydryl units can afford –S–S– linkages under the effect of oxygen [64].

Ma et al. [65] prepared a boronate-based copolymer comprising N-isopropylacrylamide, hydroxyethyl methacrylate, and (4-(hydroxymethyl)-phenylboronic acid. The presence of N-isopropylacrylamide endowed the obtained copolymer’s temperature-responsive characters. As a result, the sol-gel transformation occurs when the temperature is higher than a certain value ranging from 18 to 37 °C. The lower critical solution temperature (LCST) and upper critical solution temperature (UCST) can be controlled by the proportion of N-isopropylacrylamide. The presence of boronate moieties makes the obtained hydrogel exhibit H_2_O_2_–responsive characters. A similar strategy can also be found in another patent [66].

### 3.2. Addition of Difunctional Crosslinkers

Zhao et al. [67] prepared H_2_O_2_–responsive hydrogels using cystamine dihydrochloride as the difunctional crosslinkers. In their strategy, catecholamine-modified chitosan was prepared and used to fabricate the hydrogels. The amino groups in cystamine can react with catecholamine moieties via the formation of benzoquinone. As a result, the disulfide bonds were introduced into the matrix of the obtained hydrogels, which endowed the hydrogels with H_2_O_2_–responsive degradation abilities.

**Figure 1:**
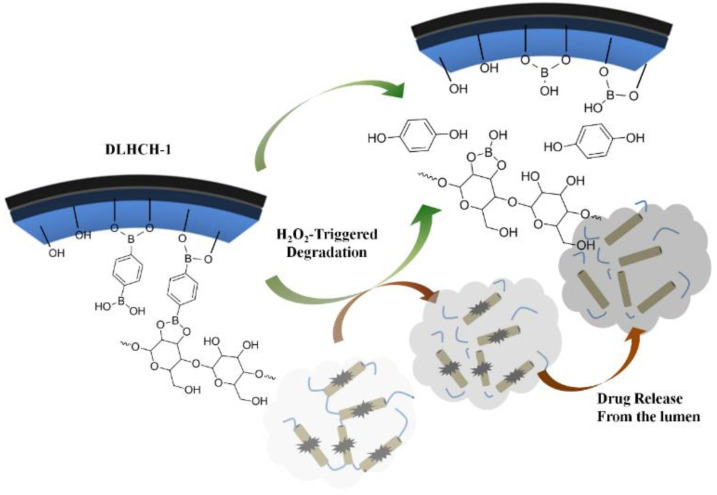
The H_2_O_2_-responsive release mechanism of the 1,4-phenylenediboronic acid-crosslinked hydrogels reported by our group (Reprinted/adapted with permission from Ref. [50]. Copyright year: 2022, copyright owner’s name: Hailei Zhang).

Our group proposed an alternative approach to synthesizing H_2_O_2_–responsive hydrogels, in which the difunctional crosslinkers and polyhydric matrix are used. For example, the arylboronic acid unit on 1,4-phenylenediboronic acid can react with the vicinal diol groups of a polysaccharide to form a chemical hydrogel with high efficiency. The B−C bond in the obtained hydrogel can be broken with the addition of H_2_O_2_ in a dose-dependent manner, which can give rise to the degradation behavior and endow the H_2_O_2_–responsive behavior to the hydrogel (Figure 1) [50]. The boronate-based difunctional crosslinkers have been also reported in other studies [68].

## 4. Application

### 4.1. Drug Delivery

Drug-loaded hydrogels have generated increasing interest in recent years because of their extraordinary advantages in versatile drug administration, ease of preparation, high biocompatibility, and improved patient compliance [69]. Moreover, the stimuli-responsiveness can be achieved by including the responsive linkages into the hydrogel networks, e.g., temperature-responsive, pH-responsive, photo-responsive, etc. H_2_O_2_ is usually involved in a variety of pathological effects in organisms, in which the aberrant expression of H_2_O_2_ is directly linked with cancer, inflammation, phyma, etc. The exploration of H_2_O_2_-responsive drug delivery systems with high selectivity and specificity has great significance for improving antitumor efficacy and preventing inflammation.

In Wan’s study, a serious of thioketal-containing microgels have been developed that show a dual responsive behavior to H_2_O_2_ and glutathione. Paclitaxel was loaded into the dual responsive thioketal microgels to achieve an antitumor preparation which is sensitive enough to the tumor-site H_2_O_2_ and glutathione levels and able to release paclitaxel accordingly. Moreover, the obtained dual responsive thioketal microgels can be degradable into biocompatible and low toxic byproducts after performing their delivering tasks. Following this method, the antitumor efficacy of chemotherapy drugs can be significantly improved by loading them into the dual responsive platform. Meanwhile, side effects can also be effectively reduced [63].

Duvall et al. [70] explored poly(propylene sulfide) nanoparticles and hydrogels as H_2_O_2_-responsive drug carriers. Generally, the conversion between sulfoxides/sulfones and hydrophobic sulfide can cause solubility changes. The hydrophobicity of poly(propylene sulfide) makes it a good candidate for loading hydrophobic drugs, similar to curcumin. After delivery in vivo, the exposure of poly(propylene sulfide) to H_2_O_2_ enables a transition to its hydrophilic form, which triggers particle swelling and H_2_O_2_-responsive drug release.

**Figure 2:**
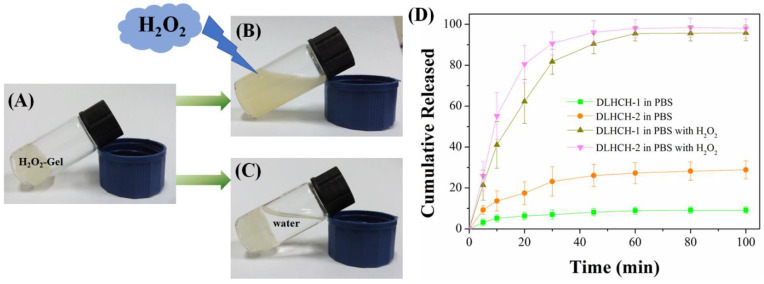
The H_2_O_2_-responsive behaviour of 1,4-phenylenebisdiboronic acid-crosslinked hydrogels reported by our group (**A**): H_2_O_2_-Gel in a transparent vial; (**B**): H_2_O_2_-Gel immersing in H_2_O_2_ aqueous solution for 100 min; (**C**): H_2_O_2_-Gel immersing in aqueous solution for 100 min; (**D**): the drug release profiles of DLHCH-1 and DLHCH-2) (Reprinted/adapted with permission from Ref. [50]. Copyright year: 2022, copyright owner’s name: Hailei Zhang).

Our group developed H_2_O_2_-Gels by using difunctional crosslinkers. A high level of H_2_O_2_ can destroy the B-C bond in the obtained hydrogels and thereby break the three-dimensional networks in H_2_O_2_-Gels. When the drug was loaded into the hydrogel, a typical H_2_O_2_-responsive behavior was easily achieved. It should be noted that hydrogels, which are diffusion-controlled drug delivery systems, usually exhibit an initial burst release. Since drug-release during the initial stage depends mostly on diffusional escape, a sudden release in a short period usually takes place. It can be regarded as an “initial burst release” [71,72]. A serious initial burst release may be toxic and give rise to suppressed bioavailability. In our study, the model drug was poured into halloysite nanotubes, a natural aluminosilicate clay mineral with a hollow tubular structure, before loading it into the matrix of H_2_O_2_-Gels (Figure 2, DLHCH-1: the therapeutic agent was pre-loaded into the halloysite nanotubes; DLHCH-2: the therapeutic agent was directly loaded into the matrix of hydrogels). Following this method, the “initial burst effect” was effectively suppressed due to the halloysite nanotubes. Our strategy provides an alternative way to design H_2_O_2_-Gels which may meet the requirements for the application of topical preparations to prevent inflammation (Figure 2) [50].

Zhen et al. focused on exploiting the boronate-based polymeric network as microneedles, which can be used as skin patches for acne vulgaris treatment. Due to the presence of boronate moieties, the antibiotic loaded composition possesses a H_2_O_2_-responsive release behavior [68].

There numerous reports focused on the development of H_2_O_2_-Gel as drug delivery systems [73,74]. However, there is still a lack of commercially available products. Further studies should pay attention to this point. Some other studies are still required. The potential side effects of the degraded products should also be investigated. Additionally, the specificity in non- enzyme systems should also be improved since the body fluid is a very complicated system.

### 4.2. Detection

H_2_O_2_ has been demonstrated to be a metabolite of many biochemical reactions. Qualitative H_2_O_2_ detection is an important tool which can be used to discover early lesions such as those present in cancer. As typical soft materials, hydrogels show a biophysical similarity to soft biological tissues and thereby possess a desirably good affinity with them. Moreover, the chemosensor moieties can be covalently linked or physically incorporated into the matrix of hydrogels to endow the product with various diagnostic applications. In past decades, studies on utilizing H_2_O_2_-Gels as detection tools were reported [75,76].

#### 4.2.1. Electrical

Tian’s group developed a hydrogel-based biosensor system by incorporating Cytochrome c into the matrix of hydrogel consisting of Fmoc-L-lysine, Fmoc-L-phenylalanine, and sodium carbonate, in which the inherent bioactive activity of Cytochrome c was retained. After treatment with H_2_O_2_, the biochemical reaction between Cytochrome c and H_2_O_2_ can result in a fast and sensitive change in the redox formal potential. Moreover, the cellular level H_2_O_2_ can be quantitively determined. Such H_2_O_2_-responsive behavior was demonstrated to exhibit excellent specificity over commonly used ions, ascorbic acid, and other ROS. The good stability and reproducibility, as well as the high specificity and sensitivity, may pave a new path to further understanding the role of H_2_O_2_ in pathological changes [18].

Li et al. prepared luminophore-incorporated polyaniline-polyacrylamide hydrogel that exhibited favorable biocompatibility and good conductivity. The obtained hydrogel showed a high detection sensitivity upon H_2_O_2_ based on an electrochemiluminescence method. The limit of detection of H_2_O_2_ was as low as 2.9 × 10^−9^ M. The detection concentration ranged from 5 × 10^−5^ to 1 × 10^−8^ M. The results indicated that the prepared H_2_O_2_-Gel can be utilized to detect cellular H_2_O_2_ [77].

The electrochemical detection of H_2_O_2_ can also be achieved by constructing Hemin-G4/Au-containing composite hydrogels. In Hou’s study [78], the excellent catalytic activity of Hemin-G4 and the high conductivity of gold nanoparticles were employed to endow the obtained hydrogels with a good electrochemical response towards H_2_O_2_. Moreover, A549 cells can adhere to the surface of the obtained hydrogels, which can be used to detect extracellular H_2_O_2_.

Electrical detection methods usually show low limit of detection (LOD) levels. The enzyme-incorporated ones exhibit good specificity, making them desirable detection tools in bioanalysis. Nevertheless, there is still a need for other detention methods that are low-cost and free of energy.

#### 4.2.2. Fluorescent

Hydrogels with a fluorescent response to H_2_O_2_ are fundamentally important in biology and pathophysiology which have practical value in early clinical diagnosis. In Yang’s study [79], an intensive chemiluminescence hydrogel was proposed by incorporating luminol and hemin into the matrix of guanosine-derived hydrogel. The obtained composite hydrogel demonstrated enzyme-like activity towards the H_2_O_2_-mediated oxidation of luminol, in which the blue light can be visibly observed.

**Figure 3:**
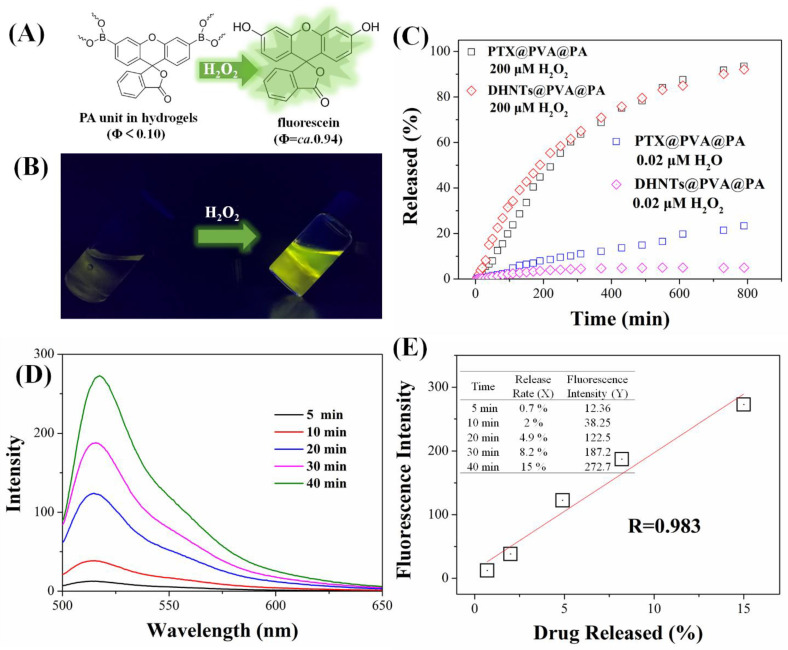
H_2_O_2_-responsive behaviors of the fluorescein-crosslinked hydrogels reported by our group: (**A**) The transformation mechanism of fluorescein-crosslinked hydrogels from arylboronates to phenols to afford fluorescein with high fluorescence in the presence of H_2_O_2_; (**B**) the changes of the hydrogel from non-fluorescent to fluorescent; (**C**) the drug release profiles in different concentration of H_2_O_2_; (**D**) the fluorescence spectra of the release medium (H_2_O_2_ = 200 μM) after addition of DHNTs@PVA@PA; (**E**) plots of fluorescence intensity vs. the drug release rate (Reprinted/adapted with permission from Ref. [51]. Copyright year: 2022, copyright owner’s name: Hailei Zhang).

Our group synthesized a novel type of fluorescein-derived fluorescence probe by installing boronic acid groups at the 3′ and 6′-positions of the xanthenone moiety in fluorescein. The obtained fluorescein derivative displayed almost no emission behavior (Φ_f_ = *ca*.0.01). After treatment with H_2_O_2_, the fluorescein derivative can be degraded into fluorescein with strong fluorescence (Φ_f_ = 0.94) [80]. The “turn-on” response in fluorescence resulted in a H_2_O_2_-detecting ability. Moreover, the two arylboronic acid groups in fluorescein derivative can react to diol units, which makes it a desirable crosslinker to fabricate the H_2_O_2_-responsive fluorescein change in the obtained hydrogels. Following this method, hydrogels with a “turn-on” fluorescence behavior upon H_2_O_2_ administration were developed (Figure 3A,B). The obtained hydrogel shows no emission behavior under the physiological H_2_O_2_ concentration, while a fast non-fluorescent to fluorescent change can be achieved under pathological H_2_O_2_ concentration [51].

Concerning the lack of an indicator to monitor the release behavior in real time for H_2_O_2_-Gels, the obtained hydrogels were also explored as drug carriers. As shown in Figure 3C, a very small amount of agent was released from DHNTs@PVA@PA under a low level of H_2_O_2_ (0.02 μM), while the 4-fold increased initial-burst amount can be tracked for PTX@PVA@PA (DHNTs@PVA@PA: the therapeutic agent was pre-loaded into the halloysite nanotubes; PTX@PVA@PA: the therapeutic agent was directly loaded into the matrix of hydrogels). The significant increase of the release rates can be observed in 200 μM H_2_O_2_ and compared to those in 0.02 μM H_2_O_2_, suggesting a typical H_2_O_2_-responsive release behavior. Figure 3D shows a remarkable increase of the fluorescence intensity over time from 5 to 40 min. The relationship between the release rate and fluorescence intensity was investigated and displayed in Figure 3E. The good relationship is quite beneficial to invisibly monitoring the drug release behavior.

#### 4.2.3. Colorimetric

**Figure 4:**
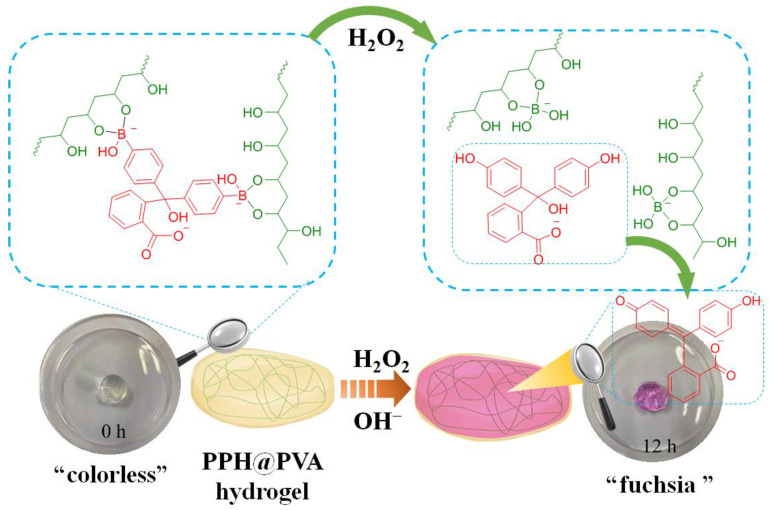
Colorimetric H_2_O_2_-responsive mechanism of phenolphthalein-crosslinked hydrogel (PPH@PVA) reported by our group (Reprinted/adapted with permission from Ref. [52]. Copyright year: 2022, copyright owner’s name: Hailei Zhang).

As a typical pH indicator, phenolphthalein solution can present a red color under an alkalescence environment based on the transformation between the phenolic hydroxyl groups and the benzoquinone unit. In our study, this colorimetric behavior was employed for the visual detection of H_2_O_2_. Boronic acid pinacol ester groups were used to substitute the hydrogel groups in phenolphthalein. As a result, the transformation between the phenolic hydroxyl groups and benzoquinone unit was blocked. Then, the phenolphthalein derivative was used to crosslink polyvinyl alcohol chains in a weak basic solution to yield a H_2_O_2_-Gel based on the dynamic covalent bonds. After treatment with H_2_O_2_ under a pathological concentration, the B–C linkage in the network can be rapidly degraded into Ar-OH groups and B-OH groups result in the formation of phenolphthalein. Since the hydrogel was prepared under an alkalescence environment, the obtained hydrogel showed a distinct colorimetric response to H_2_O_2_ (Figure 4) [52].

Yang et al. [81] reported a 2D photonic crystal-based horseradish peroxidase/bovine serum albumin (HRP/BSA) composite hydrogel. BSA and HRP was crosslinked by glutaraldehyde, in which BSA and HRP acts as a scaffold and a recognition unit, respectively. The obtained hydrogel can monitor H_2_O_2_ since HRP can selectively decompose H_2_O_2_ along with heme inactivation. The conformational change of HRP can decrease the crosslinking density and enlarge the particle spacing. As a result, the Debye ring of the 2-D photonic crystals also decreased, which provided a colorimetric response to H_2_O_2_ for the obtained hydrogel.

Compared to electrical and fluorescent methods, the colorimetric method shows a superior low cost, an easy operation, and operates free of energy. Moreover, the latter method can be used without any sophisticated or expensive analytical instruments, which makes it a desirable detection tool for actual production and daily life.

### 4.3. Glucose-Responsive

Diabetes is regarded as a serious metabolic disorder, which affects more than one-tenth of the population in the world [82,83]. Long-term high glucose levels can result in serious cardiovascular disease, renal failure, abnormal bone metabolism, and many other complications. The glucose-controlled insulin release system is usually required in the treatment of diabetes, especially for suppressing diabetes ulcers [84]. Among these systems, glucose oxidase is an important intermediary for the transformation of glucose signals into the generation of H_2_O_2_ [85]:

(1) $$\mathrm{Glucose}+O_{2}\overset{\mathrm{Glucose}\;\mathrm{oxidase}}{\to }H_{2}O_{2}+\mathrm{Gluconic}\;\mathrm{acid}$$

Therefore, with the addition of glucose oxidase, H_2_O_2_-Gel can also be used as glucose-responsive systems. Kiritoshi et al. prepared H_2_O_2_-Gel via a typical radical copolymerization of 2-methacryloyloxyethyl phosphorylcholine and triethylene glycol dimethacrylate. The obtained polymer can be easily degraded in the presence of H_2_O_2_ (974 mmol/L). In Kiritoshi’s strategy, the glucose oxidase can be loaded into the matrix of the obtained hydrogel without a loss in catalytic activity. Following this method, the obtained H_2_O_2_-Gel is also available in glucose-responsive insulin delivery systems in a concentration-dependent manner [86].

In past decades, an increasing number of studies have focused on transforming H_2_O_2_-Gels into glucose-responsive systems with the addition of glucose oxidase [64,87]. For example, Yang et al. developed a hydrogel that formed in situ by loading glucose oxidase into keratin hydrogels, which showed promising applications for the treatment of diabetic wounds [64].

### 4.4. ROS Scavengers

An excessive ROS level can raise the oxidative damage to cellular macromolecules and thereby deteriorate many inflammation-related diseases. Moreover, the ROS accumulated in a wound can induce strong inflammation and serve as a strong barrier that inhibits tissue regeneration, which is a puzzling problem for diabetic ulcers [88]. Some H_2_O_2_-Gels are promising ROS scavengers in clinical applications [35,89], especially those that have the capacity to consume H_2_O_2_ in a concentration-dependent manner [90].

Liu’s group developed a type of ROS-scavenger by using a difunctional arylboronic derivative as crosslinker [91]. The obtained H_2_O_2_-Gels can act as an effective ROS-scavenger agent to promote wound-healing by reducing the ROS level around the wound, which is quite beneficial to inhibiting aseptic inflammation. Moreover, the H_2_O_2_-Gel was also explored as a drug carrier to allow the release of therapeutic agents, such as antibacterial agents and growth factors. As a result, the H_2_O_2_-Gel supported a systemic therapy strategy to promote wound-healing in complicated infections.

Gao et al. presented a novel type of thioether-containing injectable hydrogel with H_2_O_2_-responsive behaviors, which can be used as ROS-scavengers for myocardial infarction treatment [92]. The resulting animal model showed that the obtained H_2_O_2_-Gels can significantly consume excessive ROS, accelerate angiogenesis, and improve cardiac functions.

### 4.5. Tissue Engineering

Hydrogels have been demonstrated to be a desirable candidate for cartilage regenerative substrates owing to their attractive advantages including mild reaction conditions and tunable structures. The controllable gelatinizing process, especially for the gelation time, is the key issue when hydrogel is used for articular cartilage engineering. Rahbarghazi et al. developed a library of alginate-based hydrogels crosslinked via horseradish peroxidase. Generally, horseradish peroxidase can serve as an efficient enzyme catalysis in catalyzing the conversion from H_2_O_2_ to H_2_O and O_2_ [93]:

(2) $$H_{2}O_{2}\overset{\mathrm{Horseradish}\;\mathrm{peroxidase}}{\to }2H_{2}O+O_{2}$$

As a result, the gelation time can be well-controlled based on the concentration of H_2_O_2_, which is a result of the deactivation effect of horseradish peroxidase by H_2_O_2_ [94].

It is worth noting that the tissue engineering materials are usually composed of different parts to meet the demand in terms of therapeutics. Liu’s group developed an injectable, post-trauma microenvironment-responsive, H_2_O_2_ depleting, and drug-loaded hydrogel [95], which can be used to lower the H_2_O_2_ levels in damaged brain tissue. The poly (propylene sulfide)-containing matrix endowed the obtained hydrogel with H_2_O_2_-responsive abilities. The released curcumin can further eliminate the ROS, promoting the regeneration and recovery of neurons. Following this method, a systematic and multi-channel promoting effect can be achieved, which is quite useful in tissue engineering materials.

### 4.6. Cell-Encapsulation

Hydrogel particles have been studied in the latest decade for encapsulating and delivering cells in humans, which meets many of the demands for treating diseases and producing antibodies for vaccine use. The main advantage of hydrogel-based cell-encapsulation technology is a mild reaction condition which does not threaten the lives of the enclosed cells.

Sakai et al. reported a facile approach for preparing mammalian cell-encapsulating hydrogel particles based on the horseradish peroxidase-crosslinked hydrogelation [96]. Gelatin bearing phenolic hydroxyl moieties were employed as the polymeric substrate and dissolved in a cell-suspending solution containing horseradish peroxidase. The mixed solution was then dropped into 1 mM H_2_O_2_ to form hydrogels with a spherical shape. The α-H in phenolic hydroxyl moieties usually exhibits high activity with the presence of horseradish peroxidase and H_2_O_2_ [19]. These phenomena suggested that the H_2_O_2_-responsive behavior can be used to establish a cell-encapsulation method in hydrogel particles.

In Sakai’s proposed strategy [19], the individual cell can be encapsulated into a thin hydrogel sheath. This approach can be controlled by the addition of HRP and exhibits no significant adverse effects to the encapsulated cells. Apparently, the proposed approach based on the H_2_O_2_-responsive behavior is expected to be used to extend the applications of cell encapsulation technology.

## 5. Conclusions and Perspective

Recent studies of H_2_O_2_-Gels have been reviewed in this paper. The summarized H_2_O_2_-responsive linkages and preparation methods may serve as guides for researchers who are interested in H_2_O_2_-Gels. We expect to provide guidance and designate the direction of further studies on H_2_O_2_-Gels. In combination with drug delivery, the detection of H_2_O_2_, glucose-responsive systems, ROS scavengers, tissue engineering, and cell-encapsulation, H_2_O_2_-Gels could be advanced to have much more promising applications in biomedicine. Nevertheless, commercially available H_2_O_2_-Gels, as well as official standards or criteria, are also urgently needed. There are still some limitations for this issue. The level of H_2_O_2_ usually differs among individuals, and the complexity of bodily fluids should also be considered. Therefore, it is far more difficult to establish in vitro and in vivo correlation (IVIVC), which is rarely studied but indeed remains a key issue that should be focused on. On the other hand, costly proteinases seriously limit large-scale production. Non-proteinase ones usually suffer from other problems such as stability, complexity in the synthesis approach, the potential toxicity of the degraded by-products, etc. Thus, longer-duration studies are encouraged to conduct deeper investigations to make H_2_O_2_-Gels “over the counter”.

## Figures and Tables

**Table 1:** Summary of responsive mechanism to H_2_O_2_.

No.	Mechanism	Literature
1	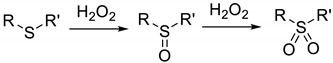	[31,32]
2	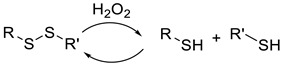	[33,34]
3	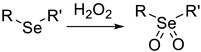	[35]
4	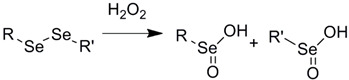	[36]
5	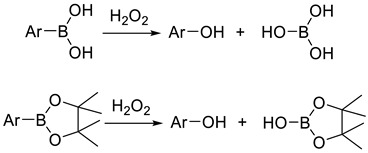	[37]
6	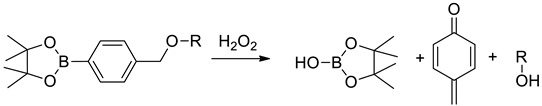	[38]
7	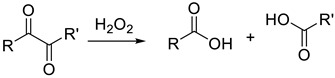	[39,40]
